# Synthesis Mechanisms, Structural Models, and Photothermal Therapy Applications of Top-Down Carbon Dots from Carbon Powder, Graphite, Graphene, and Carbon Nanotubes

**DOI:** 10.3390/ijms23031456

**Published:** 2022-01-27

**Authors:** Wenquan Shi, Qiurui Han, Jiajia Wu, Chunyu Ji, Yiqun Zhou, Shanghao Li, Lipeng Gao, Roger M. Leblanc, Zhili Peng

**Affiliations:** 1 School of Materials and Energy, Yunnan University, Kunming 650091, China; wqshi@mail.ynu.edu.cn (W.S.); hanqr@mail.ynu.edu.cn (Q.H.); 12019101477@mail.ynu.edu.cn (J.W.); jichunyu@mail.ynu.edu.cn (C.J.); gaolp@mail.ynu.edu.cn (L.G.); 2 Advanced Computing Center, Materials Genome Institute, Yunnan University, Kunming 650091, China; 3 Department of Chemistry, University of Miami, 1301 Memorial Drive, Coral Gables, FL 33146, USA; yxz431@miami.edu (Y.Z.); rml@miami.edu (R.M.L.); 4 MP Biomedicals, 9 Goddard, Irvine, CA 92618, USA; Shanghao_li@hotmail.com

**Keywords:** carbon dots, carbon nano powders, graphite, graphene, carbon nanotubes, photothermal therapy

## Abstract

In this study, top-down syntheses of carbon dots (CDs) from four different carbon precursors, namely, carbon nano powders, graphite, graphene, and carbon nanotubes, were carried out. Systematic study demonstrated that the optical properties and surface functionalities of the CDs were quite similar and mainly influenced by the synthesis method, while the sizes, morphologies, chemical compositions, and core structures of the CDs were heavily influenced by the carbon precursors. On the basis of these studies, the formation processes and structural models of these four top-down CDs were proposed. The cell cytotoxicity and photothermal conversion efficiency of these CDs were also carefully evaluated, demonstrating their potential applications in photothermal therapy.

## 1. Introduction

Carbon dots (CDs), a class of zero-dimensional nano materials with sizes generally less than 10 nm, have aroused attention from scientists all over the world ever since their early discovery [1,2]. Thanks to their superior properties, CDs have been successfully applied in many fields, such as in sensing [3,4], drug delivery [5,6], gene therapy [7], biological imaging [8], photocatalysis [9,10], and electrocatalysis [11,12]. Since CDs were first discovered via arc discharge and lately promoted by laser ablation [2], CDs were generally prepared by cracking bulk carbon materials in the early days (top-down synthesis). In 2013, Zhu et al. successfully synthesized CDs by a hydrothermal method with citric acid and ethylenediamine for the first time [13], which marked the transformation of carbon precursors for CDs synthesis from bulk, inorganic carbon materials to small, organic molecules (bottom-up synthesis). So far, the countless methods for CDs synthesis can be classified into two categories: top-down and bottom-up approaches. The top-down approach is to decompose macroscopic carbon materials into nano-sized particles under harsh conditions such as arc discharge, laser ablation, and acid oxidation exfoliation. On the other hand, the bottom-up approach employs small molecules as the carbon sources and synthesis can be realized via microwave-, hydrothermal-, or solvothermal-assisted treatments [14].

Compared to the bottom-up approach, CDs synthesized from a top-down approach demonstrate several significant advantages. One obvious advantage of the top-down strategy is the ability to synthesize CDs with controllable sizes. Since CDs are prepared via the breaking down of bulk carbon precursors, the sizes of the CDs formed can be controlled by tuning the “cracking force” applied. For instance, Pang and co-workers obtained CDs with different sizes by adjusting the potential in the process of the electrochemical etching of carbon fibers [15]. On the other hand, size control is relatively hard for CDs from the bottom-up approach, since the polymerization–carbonization process for CDs formation is hardly controllable under high temperature and pressure. Furthermore, thanks to the use of carbon precursors with a single composition and clear structure (i.e., graphite, graphene), most CDs from the top-down approach generally have a clear core structure and composition. Lastly, it should not be ignored that CDs prepared by the top-down approach from bulk carbon materials generally have clear surface functionalities (i.e., hydroxyl and carboxyl groups) [16], which are easier to modify further. Thus, various functionalities, drug molecules, fluorophores, and even antibodies could be introduced onto the surface of top-down CDs to endow them with the desired properties and functions [17]. Exploiting these advantages, top-down CDs are very promising and have been widely applied for various purposes, such as photocatalysis [18], light emitting devices [19], bioimaging [20], and biomedicine. In addition to these studies, top-down CDs have also shown great potential for applications in solar cells [21], biosensing (i.e., ion detection), and food analysis [22].

As various studies have demonstrated, the properties of CDs determine their applications, and the properties of CDs are heavily dependent on the synthesis methods and carbon precursors (especially in the case of top-down CDs). However, there have been less studies reported specifically focusing on the influences of the carbon precursors on the properties of top-down CDs, lagging far behind their applications. Herein, we report the first systematic study on the relationship between the intrinsic characteristics of the carbon precursors and the properties of the top-down CDs formed. In this work, four carbon allotropes, namely, carbon nano powders (CNPs), graphite, graphene, and carbon nanotubes (CNTs), have been selected as the carbon precursors for the synthesis of top-down CDs under the same reaction condition. Moreover, the structural, chemical, physical, and biological properties of the CDs derived from these four precursors have been carefully evaluated and compared. Our results show that top-down CDs could be successfully synthesized from any of these four materials, although with different yields. Interestingly, these CDs, despite being derived from different carbon precursors, behaved similarly in their optical characteristics, thermal stability, and photothermal conversion efficiency. Moreover, there were distinct differences among these four CDs, obviously influenced by the structural differences of their corresponding carbon precursors. For instance, the sizes and surface functionalities of these four CDs have their own unique characteristics. Encouragingly, despite these differences, all four CDs demonstrated an excellent biocompatibility and photothermal conversion efficiency, which shows their great potential for applications in photothermal therapy. The systematic analysis and comparison of the basic properties of CDs derived from different carbon precursors should provide some important references for the further development of top-down CDs, especially in a rational way.

## 2. Results and Discussion

### 2.1. CDs Synthesis Optimization

**Figure 1:**
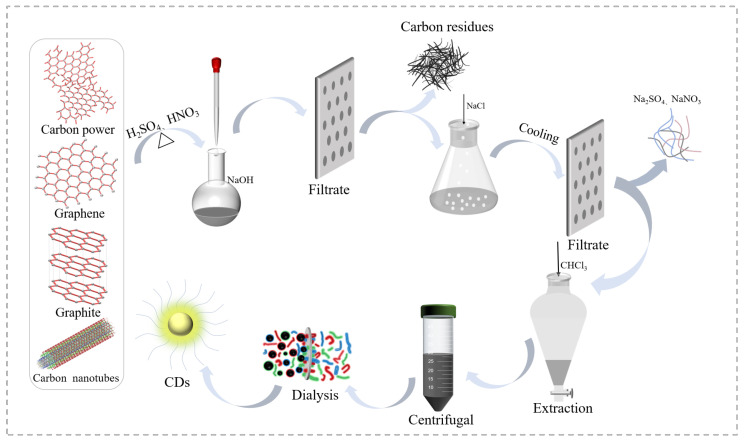
Schematic diagram of the CDs synthesis process via strong oxidative acids treatment.

To investigate the influences of carbon precursors on the structure and property of the top-down CDs formed, four commonly seen carbon precursors (CNPs, graphite, graphene, and CNTs) were selected, which included three-dimensional (3D), 2D, and 1D carbon materials. We selected the strong acid-assisted oxidative method for the synthesis of CDs, since the method is known for (1) its ability to generate rich surface functionalities (i.e., carboxyl, hydroxyl) on CDs, which could avoid an extra step for the surface passivation and (2) the method is easy to operate and does not need complex equipment such as a laser, arc, or electrochemical system. In this reaction, the key step is the breakdown of the carbon precursors into small particles under the synergistic physical (i.e., stirring and refluxing) and chemical (the oxidization of carbon bonds) forces (Figure 1). Thus, any factors that could help this process would be beneficial for the synthesis of CDs. As such, the type and quantity of acids, reaction temperature and time, as well as stirring and refluxing efficiency all play decisive roles in the yield and quality of the CDs.

To optimize the above-mentioned reaction parameters, we first used CNPs as the standard carbon precursor to probe this reaction, in which CNPs (100 nm) were decomposed into nanoscale carbon particles via vigorous stirring, refluxing, and acid stripping followed by thorough oxidization in the presence of sulfuric and nitric acids, generating CDs with rich oxygen-containing functional groups (i.e., -OH, -COOH, C–O–C) on the surfaces. Since acids play very important roles (carbon precursor stripping and oxidization), we first investigated the influence of the ratio of sulfuric and nitric acid on the synthesis of the CDs. Interestingly, our study showed that varying the ratio of sulfuric and nitric acid had little influence on the reaction; all the ratios we tested yielded similar results (Table 1, rows 1–4). However, if only sulfuric acid was used, CDs failed to form (Table 1, row 5). This might be attributed to the fact that nitric acid has a stronger oxidation ability than that of sulfuric acid; further, the oxidization capabilities of the mixture of sulfuric and nitric acids is better known than those of the two individually.

Next, we also probed the effects of reaction temperature and time on the yield of CDs. Our study showed that as long as the temperature was above 110 °C (high enough to generate efficient refluxing), further increasing the temperature did not improve the yield (Table 1, row 3 vs. 6); thus, 110 °C was selected as the standard reaction temperature. As for the reaction time, 15 h of refluxing seemed the optimal time required. Any time less than 15 h would decrease the yield (Table 1, row 7), while further increasing the time did not enhance the reaction yield significantly (Table 1, row 8). Interestingly, the yields of this reaction also relied heavily on the scale of the reaction. Our investigation revealed that when CNPs were less than 250 mg, the yields of CDs were generally high (about 43–47%) under the optimized conditions. However, when we doubled the scale of the reaction (500 mg of CNPs), the yield of CDs was significantly decreased (Table 1, row 9). Considering the fact that efficient stirring is essential for this reaction, we attributed the low yield to the inefficient stirring as the large-scale reaction became very sticky and difficult to stir.

With the above-mentioned optimizations, we established the standard synthesis in which 250 mg of carbon precursor was treated with mixture of sulfuric (9 mL) and nitric (3 mL) acids under vigorous stirring and refluxing at 110 °C for 15 h. We were able to successfully synthesize CDs from three other carbon precursors (graphite, graphene, and CNTs) with this set of optimized parameters, although with slightly lower yields (Table 1, rows 10–12). Furthermore, it seemed that the parameters optimized for CNPs were also the best conditions for the other three precursors. Any deviation from these conditions would result in a decreased yield of CDs. For instance, the yield of CDs produced from graphene was significantly reduced by either shortening the reaction time or lowering the temperature (Table S1, rows 1–2). Increasing the scale of the synthesis also had a negative effect on the yield of graphene-derived CDs (Table S1, row 3). For better clarity, we denote CDs derived from CNPs, graphite, graphene, and CNTs as C-CDs, G-CDs, GR-CDs, and CNT-CDs, respectively.

### 2.2. Morphology Characterization

**Figure 2:**
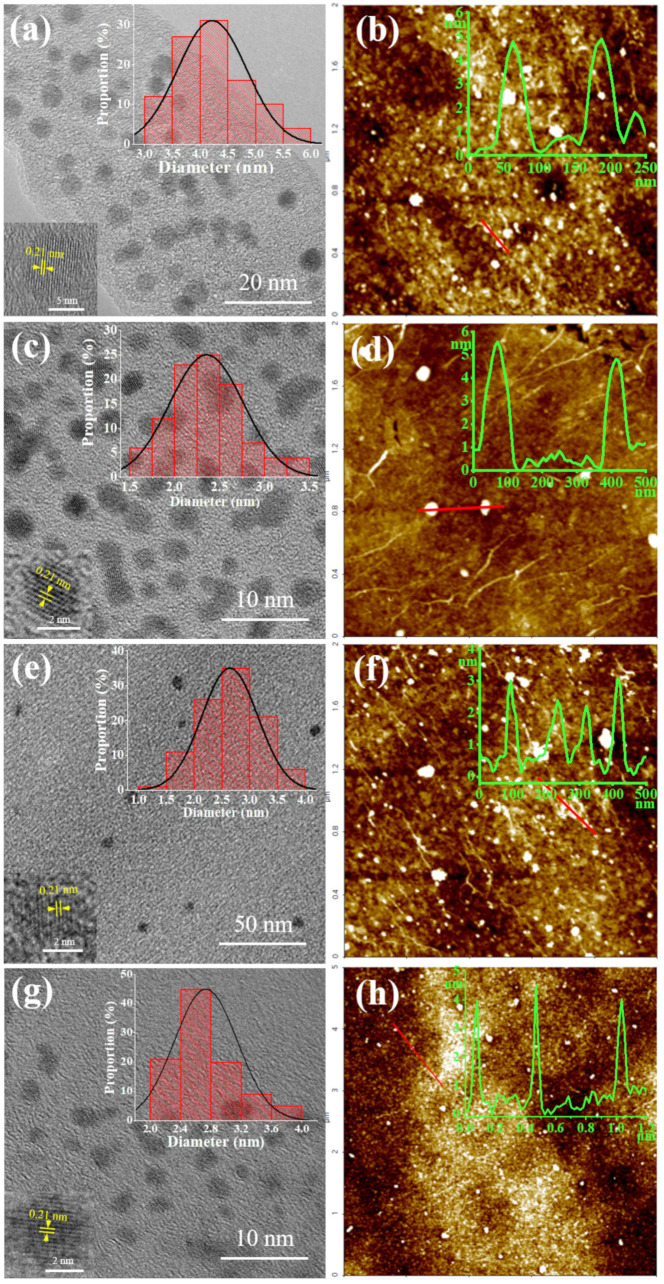
TEM and AFM images of C-CDs (**a**,**b**), G-CDs (**c**,**d**), GR-CDs (**e**,**f**), and CNT-CDs (**g**,**h**) and their particle size distribution (insert, upper right) and high-resolution lattice images (insert, lower left); the TEM histograms were based on more than 100 particles.

To further analyze the morphological, optical, chemical, and structural properties, characterizations of the CDs by TEM and AFM microscopy, UV–Vis and fluorescence, FTIR and XPS, XRD and Raman spectroscopy, as well as TGA and zeta potential, were performed. TEM and AFM microscopy were first applied to explore the morphologies and structural characteristics of these CDs (Figure 2). The TEM images of the CDs (Figure 2, left column) show that they were all well-dispersed spherical particles with no obvious agglomerates, which might be attributed to their negatively charged surfaces as indicated by their zeta potentials (Table 2**,** row 4). There are noticeable lattice fringes with a lattice spacing of 0.21 nm in all four samples (Figure 2, left column, lower left insets), which coincides with the (100) lattice planes of graphite carbon [23], indicating that they all had a certain degree of graphitic cores. These particles ranged from 1.5 to 6.0 nm in diameter with an average of 4.2 ± 0.6, 2.4 ± 0.4, 2.6 ± 0.5, and 2.7 ± 0.4 nm for the C-CDs, G-CDs, GR-CDs, and CNT-CDs, respectively (Figure 2, left column, upper right insets). From AFM microscopy (Figure 2, right column), the thicknesses of the C-CDs, G-CDs, GR-CDs, and CNT-CDs were determined to be 4.5, 5.0, 2.5, and 4.0 nm, respectively. As can be seen, the size of the C-CDs was much larger than that of the G-CDs and GR-CDs, which could be attributed to the fact that the breaking of covalent bonds (for CNPs) is much more difficult than noncovalent pi–pi stacking (for graphite and graphene). From AFM, the thicknesses of the G-CDs (5.0 nm) were twice that of the GR-CDs (2.5 nm), indicating the G-CDs had more layers of graphene than the GR-CDs. Collectively considering the characterizations from TEM and AFM, the C-CDs and GR-CDs could be regarded as spherical particles while the G-CDs and CNT-CDs could be regarded as ellipsoidal ones.

### 2.3. Optical Characterization

**Figure 3:**
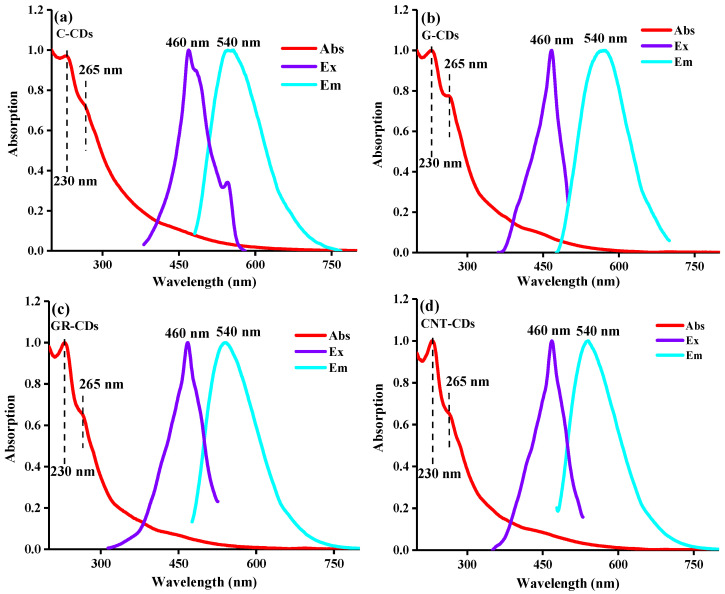
UV–Vis absorptions, PL excitations (emissions monitored at 540 nm), and PL emissions (excited by 460 nm) spectrum of CDs: (**a**) C-CDs; (**b**) G-CDs; (**c**) GR-CDs; and (**d**) CNT-CDs.

Following the morphology study, we carefully compared the optical features of these four CDs. As can be seen from Figure 3, all the CDs demonstrated very similar absorptions, excitations, and emissions, indicating that they have very similar optical features despite their different carbon precursor origins. Specifically, the UV–Vis spectra show that all the CDs have obvious broad absorptions around 200–300 nm (Figure 3, red curves), which could be attributed to the π–π* transition of C=C (230 nm) and the *n*–π* transition of C=O (265 nm) [24]. In the excitation spectrum, all the CDs have the best excitations at 460 nm (Figure 3, purple curves), resulting in maximum emissions at 540 nm (Figure 3, cyan curves), which are typical for CDs.

**Figure 4:**
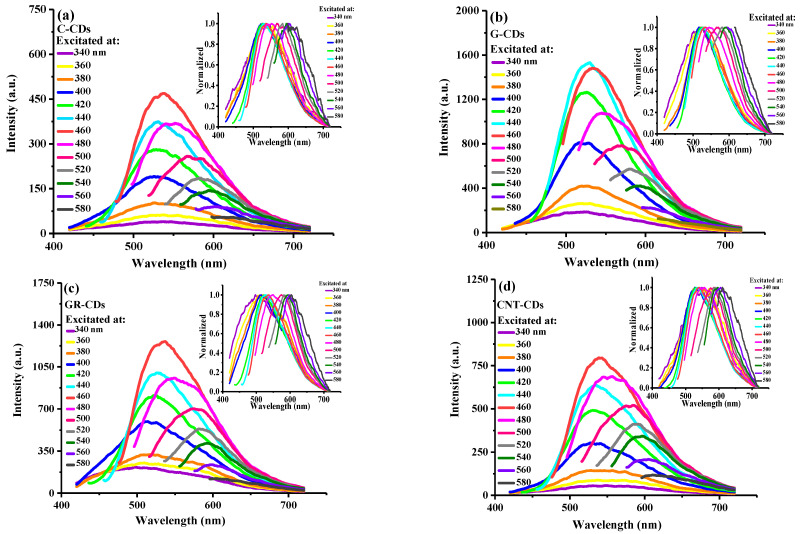
Fluorescence spectra of 0.5 mg/mL aqueous dispersions of (**a**) C-CDs; (**b**) G-CDs; (**c**) GR-CDs; and (**d**) CNT-CDs with excitation wavelengths ranging from 340 nm to 580 nm; insets: the normalized spectra.

As CDs are known for their excitation wavelength-dependent emissions, we also carefully evaluated and compared this feature of our CDs. As can be seen, all CDs indeed demonstrated this typical excitation wavelength-dependent emission behavior (Figure 4). Specifically, as the excitation wavelengths gradually increased from 340 to 580 nm, the corresponding emission peaks shifted from 520 to 630 nm, which could be clearly observed in the normalized spectra (Figure 4, insets). We further tested the fluorescence decay profile of the four CDs in aqueous solutions, and our results show they exhibited similar profiles (Figure S1). The fluorescence lifetimes for the C-CDs, G-CDs, GR-CDs, and CNT-CDs were calculated to be 3.73, 3.82, 3.88, and 4.29 ns, respectively; these τ_ave_ values are consistent with those reported in the literature [25]. Overall, the fluorescence lifetimes of the four CDs are comparable, and the slight difference could be attributed to the differences in their surface states, which may be related to their sizes and surface charges. Notably, all the CDs demonstrated an excellent PL stability: the emission wavelength and intensity of the aqueous CDs dispersions hardly changed after two months of storage under ambient conditions (Figure S2). In short, the studies shoedn that although our CDs were derived from different precursors and their morphologies are different, they demonstrated very similar optical properties. This phenomenon could be attributed to the fact that although our carbon sources are diverse, they all have similar π-conjugated domains; the optical properties of these CDs are mainly determined by the π-conjugated domains [26,27,28], and thus, the four CDs demonstrated very similar optical properties.

### 2.4. Chemical Compositions

**Figure 5:**
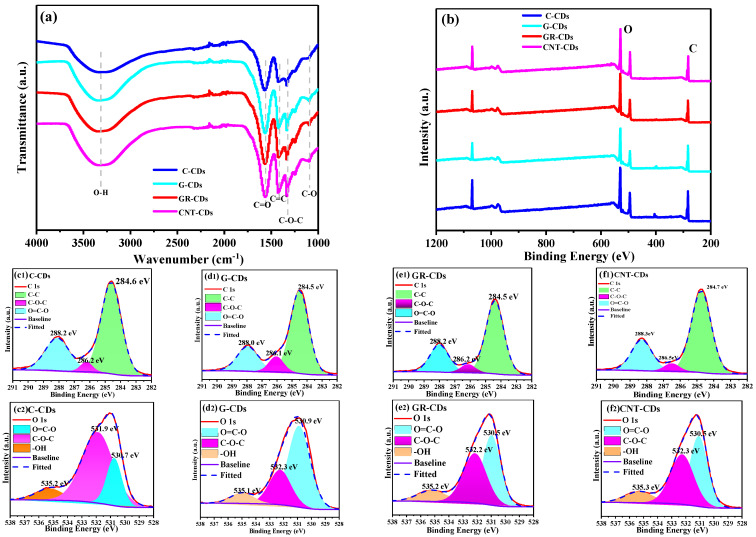
Analysis of surface functionalities of CDs: FTIR spectrum (**a**); XPS survey (**b**); and C 1 s spectra and O 1 s spectra of C-CDs (**c1**,**c2**), G-CDs (**d1**,**d2**), GR-CDs (**e1**,**e2**), and CNT-CDs (**f1**,**f2**).

Following the comparison of the optical properties, we then carefully characterized and compared the chemical compositions of our CDs via FTIR and XPS. As shown in the FTIR spectra, all four CDs also behaved very similarly, giving similar characteristic peaks in the range of 1000–4000 cm^−1^ (Figure 5a). Specifically, the bands at 3332 cm^−1^ correspond to the O–H stretching vibration. Those at 1567 and 1097 cm^−1^ could be attributed to the C=O stretching vibration and C–O vibration band [29], respectively, both of which belong to the carboxyl functionalities. The bands at 1422 cm^−1^ could be ascribed to C=C stretching and are probably from the graphitic core of CDs. Interestingly, the C-CDs, compared to the other three CDs, demonstrated a relatively weak peak at this position, which was probably due to their amorphous carbon powder origin. Vibration bands at 1333 cm^−1^ were assigned to C–O–C asymmetric stretching vibration, which probably originated from the epoxy functionalities. As can be seen, there are no obvious differences in the FTIR spectra of the four CDs, which could be attributed to the fact that they were all synthesized from strong oxidizing acidic conditions, rendering them with rich oxygen-containing functionalities such as carboxyl, hydroxyl, and epoxy.

We also compared the chemical compositions of these CDs using XPS spectra. From the full scan survey spectra of the four samples, it is clear that a rich presence of carbon (C 1 s, 284 eV) and oxygen (O 1 s, 532 eV) is present at the surfaces of the CDs (Figure 5b), which indicates that the carbon precursors have been heavily oxidized by the acids. Interestingly, the degree of oxidation varied for these CDs as revealed by the different ratios of C/O from the XPS survey (Table 2, row 1–2), which we attributed to the fact that the difficulty of oxidizing the carbon precursors is not the same. Specifically, the C-CDs contain 66.5% carbon and only 33.5% oxygen, the oxygen content being significantly lower than that of the other three samples. Thus, the CNPs are much more difficult to oxidize than the other three carbon precursors since the oxidation of CNPs requires not only the stripping off of large particles but also the breaking of carbon–carbon covalent bonds.

We further analyzed the high-resolution C 1 s and O 1 s spectra of the four samples; all the CDs have very similar spectra (Figure 5c–f). Taking the C-CDs as an example, the C 1 s spectrum could be deconvoluted into three binding energies, whose peaks center at 284.6, 286.2, and 288.2 eV, corresponding to the presence of C–C, C–O–C, and O=C–O, respectively (Figure 5(c1)), while the distinct peaks at 530.7, 531.9, and 535.2 eV in the O 1 s spectrum correspond to O=C–O, C–O–C, and -OH groups, respectively (Figure 5(c2)). From the high-resolution XPS spectrum, we can see that the C-CDs had a rich presence of functionalities such as carboxyl, hydroxyl, and epoxy at the surface, which is in high accordance with the analysis from the FTIR spectrum. Further analysis showed that the other three CDs exhibited functional groups very similar to those of the C-CDs based on their corresponding high-resolution XPS spectra. Based on the FTIR and XPS analyses above, we may conclude that the difficulty of oxidizing the four precursors varied, and thus the oxygen contents of the CDs were different. However, since all the CDs were oxidized by the same oxidative acidic environment during the reactions, the nature and type of their surface functionalities were very similar.

To obtain quantitative information about the presence of surface carboxyl groups of these CDs, we also carried out straightforward acid–base titrations to determine the contents of carboxyl groups. The contents of carboxyl groups were determined to be 6.5, 4.3, 4.4, and 8.5 mmol/g for the C-CDs, G-CDs, GR-CDs, and CNT-CDs, respectively (Table 2, row 3). Interestingly, the content of carboxyl groups (8.5 mmol/g) in the CNT-CDs was significantly higher than that of the other three samples. This might be attributed to the unique structure feature of CNTs, which could form carboxyls all over the surface under stronger acid oxidization. Further, the high content of carboxyls on the CNT-CDs matched well with their high surface negative charges as indicated from their zeta potential (Table 2, row 4). The carboxyl content (6.5 mmol/g) of the C-CDs was second only to that of the CNT-CDs, while the G-CDs and GR-CDs had very similar carboxyl contents, with the G-CDs having the lowest of the four. It is well known that when graphite or graphene are oxidized, carboxyl groups tend to form on the edges and hydroxyl and epoxy groups are generally found at the planar surfaces [30]. This might be the reason that, although both the G-CDs and GR-CDs had high surface oxygen contents (Table 2, row 2), their surface carboxyls were relatively low (Table 2, row 3).

### 2.5. Thermal Stabilities

**Figure 6:**
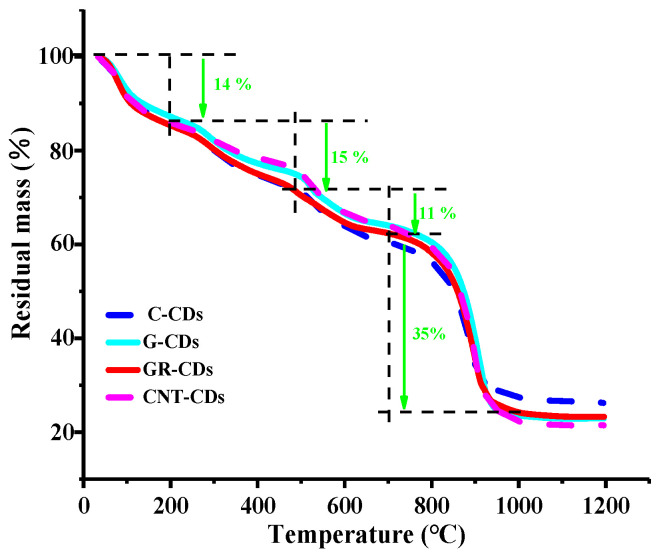
Thermogravimetric analysis curves of CDs at a heating rate of 20 °C/min under N_2._

To investigate and compare the thermal stabilities of the CDs, thermogravimetric analysis (TGA) was carried out. As can be seen (Figure 6), the weight loss tendency of the four samples was very similar, and the weight decrease process could be divided into four stages. Overall, in the first three stages, the weight losses of the CDs were all smooth and slow, while in the fourth stage, they all had a sharp weight loss. Specifically, the four CDs had only a 14% weight decrease in the first stage (0–200 °C), which could be attributed to the evaporation of adsorption water and small molecules connected via weak hydrogen bonds in the samples [31]. In the second stage (200–450 °C), there was about a 15% total weight reduction for the samples, possibly due to the degradation of -COOH of the CDs [32]. During the third stage (450–700 °C), the weight loss was only about 11%, which may have been due to the gradual decomposition of other organic functional groups (i.e., -OH) on the surface of the CDs. Significant weight reductions occurred in the last stage (temperature range 700–1000 °C), the weight losses of which could be attributed to the depletion of residual carbons under high temperature. Interestingly, a further increase of temperature (1000–1200 °C) did not change the residual masses of the samples, probably due to the fact that the remaining materials were mainly composed of charred carbon cellulose [33].

Quantitatively speaking, there was 26, 23, 24, and 21% of residue left for the C-CDs, G-CDs, GR-CDs, and CNT-CDs, respectively. The residual amount of the C-CDs was slightly higher than that of the other three samples, which corresponds well with the fact that the ratio of C/O in the C-CDs was higher (Table 2, row 1–2) according to the XPS measurement. As expected, the residual amounts for the G-CDs and GR-CDs were quite similar, as their precursors have very similar structural features. The residual weight of the CNT-CDs was the smallest, which indicates the CNT-CDs had the highest degree of oxidization among the four during synthesis. The TGA study showed that, although the CDs were synthesized from four different carbon precursors, the nature and type of their surface functionalities were very similar, which is in accordance with the FTIR and XPS analyses above. Moreover, the trivial differences in their weight losses during each stage and residual masses were most likely due to the slight differences in the oxidation difficulty of the four precursors as previously discussed.

### 2.6. XRD and Raman

**Figure 7:**
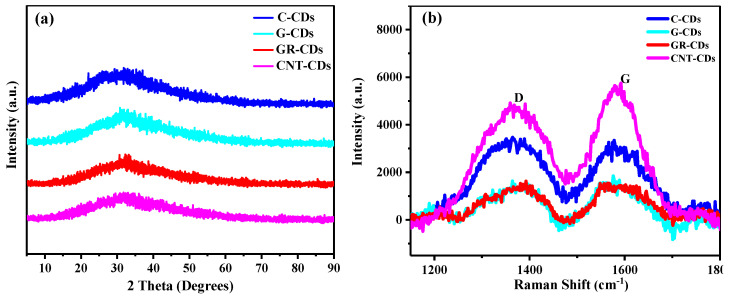
XRD patterns (**a**) and Raman spectra (**b**) of CDs.

To further probe the structural and chemical features of the CDs, we also carried out XRD and Raman analyses. As can be seen, all the CDs presented wide peaks near 32 degrees (Figure 7a), which could be attributed to the fact that the CDs formed highly disordered structures, instead of pristine graphene-like structures [34]. Moreover, the increased disorderliness was most likely due to the oxidization of the carbon–carbon bonds in the precursors. The investigation from Raman spectroscopy also resulted in similar conclusions. As can be seen, there are two obvious characteristic peaks near 1380 and 1580 cm^−1^ (Figure 7b), representing the D- and G-band, respectively. The D-band is generally referred to as the disordered band or defect band because of the oscillating bond vibration of carbon atoms on the edge plane of disordered graphite, while the G-band corresponds to the in-plane tensile vibration E_2g_ mode of graphite, which is related to sp^2^ carbon atoms [35]. The intensity ratio of the D- and G-bands (I_D_/I_G_) can be used to evaluate the degree of disorderliness/structural defect of carbon materials [36].

In our work, the ratio of D/G (I_D_/I_G_) in the C-CDs was 1.06, higher than that of the G-CDs (I_D_/I_G_ = 0.87), GR-CDs (I_D_/I_G_ = 0.88), and CNT-CDs (I_D_/I_G_ = 0.85), which means the C-CDs have a much higher disorderliness than that of the other three CDs. Collectively considering the nature of the precursors of the CDs (disordered CNPs for the C-CDs, and highly ordered graphene derivatives graphite, graphene, and CNTs for the G-CDs, GR-CDs, and CNT-CDs, respectively), the differences in the I_D_/I_G_ values of the four samples could be related to the intrinsic structural differences of the carbon precursors (the Raman spectrum of the raw materials is presented in Figure S3). Specifically, the C-CDs demonstrated the highest I_D_/I_G_ value, which could be attributed to the disordered structure features inherited from CNPs. Meanwhile, the I_D_/I_G_ values for the G-CDs, GR-CDs and CNT-CDs were very close and much smaller than that of the C-CDs, probably due to the fact that the carbon precursors of the three CDs can all be regarded as graphene derivatives [37], which are structurally much more ordered than CNPs. Based on the above discussions, we may conclude that the intrinsic nature of the carbon precursors could also determine some of the structural properties of the CDs formed.

### 2.7. Formation Process and Structure Models of the CDs

Generally speaking, in our approach of CDs synthesis, the bulky carbon precursors were broken down into small particles that were simultaneously functionalized to form the CDs (Figure 1). Since all four precursors went through the same reaction conditions (strong oxidative acids treatment), the generated CDs demonstrated the same type of surface functionalities (i.e., carboxyl, hydroxyl, and epoxy) as previously discussed. However, due to the difference of the precursors, the degree of oxidization was different for these samples; thus, their contents of functionalities were different as well as their carbon/oxygen ratios. Furthermore, the difficulty of breaking down the four precursors was quite varied, and thus the formed CDs demonstrated quite different sizes and morphologies. It is worth noting that the core structures of the CDs were basically determined by the intrinsic nature of their precursors as revealed by our studies.

**Figure 8:**
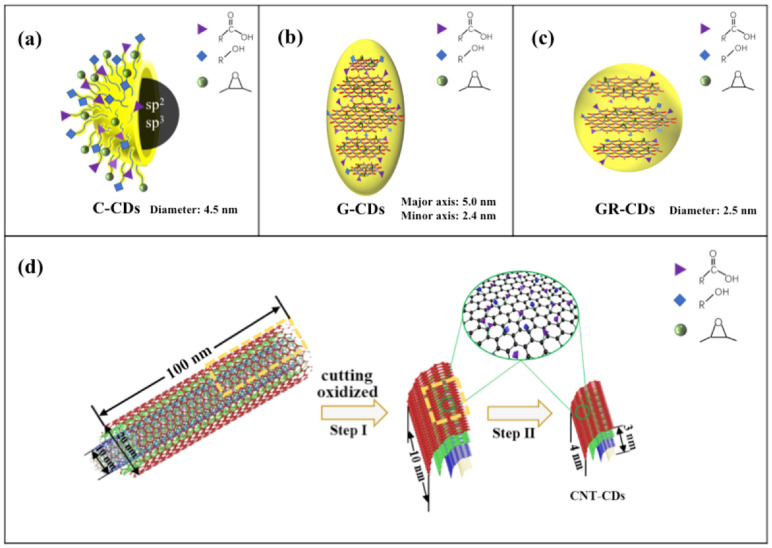
Proposed structural models for C-CDs (**a**), G-CDs (**b**), and GR-CDs (**c**), which show the size, spatial configuration, and surface functionalities; proposed synthesis process and structural model for CNT-CDs (**d**). The figures were not drawn to scale.

Collectively considering the abovementioned morphological, optical, chemical, and structural characterizations, the structural models for the four CDs are proposed (Figure 8). The C-CDs could be considered as spherical particles (diameters around 4.5 nm) with amorphous cores; on the surface, they have abundant oxygen-containing functionalities, with carboxyls being the largest group (Figure 8a). The G-CDs could be regarded as ellipsoidal particles, with diameters of 5.0 and 2.4 for the major axis and minor axis, respectively. These particles consisted of roughly five to six layers of graphene, which function as the core (Figure 8b). On the other hand, the GR-CDs could be considered as spherical particles with an average diameter of 2.5 nm. They consisted of roughly three layers of graphene as the core (Figure 8c). Although the core structures of the G-CDs and GR-CDs were different, their surface functionalities were quite similar to that of graphene oxide [30]: the carboxyl groups were mainly distributed on the edges, while the hydroxyl and epoxy groups were mainly located at the planar surfaces among the graphene layers (Figure 8b,c). As a result, although both the G-CDs and GR-CDs had a higher oxygen content than that of the C-CDs, their surface carboxyl groups were less than that of the C-CDs (Table 2).

According to the TEM and AFM characterizations, the CNT-CDs appeared as ellipsoidal particles, with diameters of 4.0 and 2.7 for the major axis and minor axis, respectively. Thus, we speculate that the CNT-CDs were the broken pieces of the CNTs, which still maintained the original multi-walled structural feature (Figure 8d). The CNTs we used had a radius of 10–20 nm and a length of 20–100 nm, which could be cut into medium-sized pieces under vigorous stirring with high temperature and concentrated oxidative acids (Figure 8d, step I). Indeed, we observed such medium-sized, broken multi-walled CNT pieces in the unpurified CNT-CDs samples. As can be seen, the average length and width of these pieces were about 8.0 and 3.5 nm, respectively (Figure S4). Under continuous reaction, these pieces could be further broken down to form the final product CNT-CDs (Figure 8d, step II), which were broken pieces of CNTs still maintaining some degree of the original multi-walled structural feature. Since these particles had large surface areas exposed for oxidization, they had the highest content of carboxyl groups among the four CDs (Table 2).

### 2.8. Photothermal Efficiency

**Figure 9:**
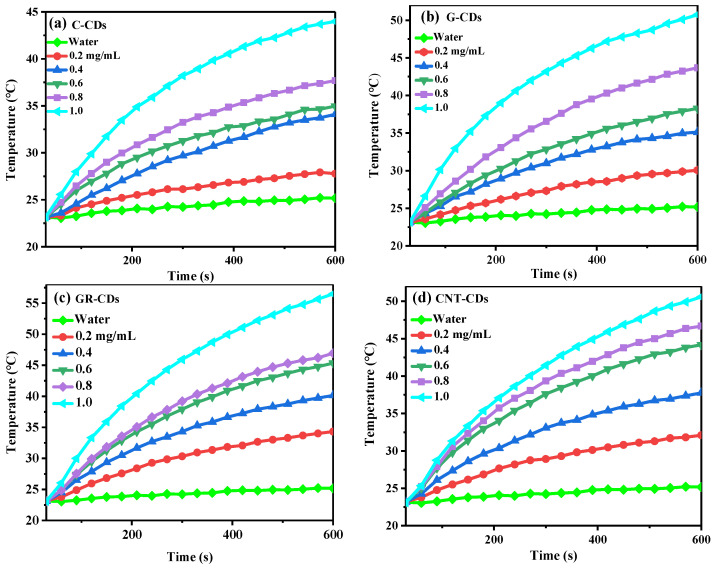
Temperature elevation of CDs dispersions of different concentrations irradiated by an 808 nm laser for 10 min: (**a**) carbon nanopowder derived CDs (C-CDs), (**b**) graphite derived CDs (G-CDs), (**c**) graphene derived CDs (GR-CDs) and (**d**) carbon nanotubes derived CDs (CNT-CDs).

Considering the fact that our CDs demonstrated relatively low PL efficiency (QYs were 2.88, 0.40, 0.35, and 0.11% for the C-CDs, G-CDs, GR-CDs, and CNT-CDs, respectively), which is typical for top-down CDs, we tested the potential of our CDs for photothermal treatment. The four samples all demonstrated excellent photothermal conversion capability, which was concentration dependent (Figure 9). Without CDs, the temperature of pure water was only increased by 2.0 °C from r.t. after 10 min of continuous irradiation by an 808 nm laser. In contrast, the solution temperatures were significantly increased in the presence of CDs after laser irradiation, and the degree of temperature increase was positively correlated with the concentrations of CDs dispersion. Specifically, when 0.2–1.0 mg/mL of C-CDs, G-CDs, GR-CDs, and CNT-CDs were dispersed, solution temperatures could be raised by 4.7–20.8, 8.9–27.9, 11.4–33.7, and 8.9–28.1 °C, respectively (Figures S5–S8). It is generally accepted that cancer cells can be effectively killed when maintained at 42 °C for 15–60 min. However, if the temperature exceeds 50 °C, the time needed to kill cancer cells could be reduced to 4–6 min [38]. In our study, CDs dispersions with concentrations of 0.2 and 0.4 mg/mL could raise the temperature by 11.5 and 17.4 °C, respectively. Considering the fact that normal body temperature is 36–37 °C, this means that after 10 min of irradiation, CDs dispersions of 0.2 and 0.4 mg/mL could effectively kill cancer cells in a short amount of time.

To better compare the photothermal capability of the CDs, we also calculated their photothermal conversion efficiencies (η, calculated with 0.4 mg/mL samples). The η_808_ values of the C-CDs, G-CDs, GR-CDs and CNT-CDs were determined to be 39.9, 45.0, 45.7, and 24.3%, respectively, which are comparable to those of metal oxides (η_808_ = 20–55%) and metal sulfides (η_808_ = 24–48%) [39].

### 2.9. Cell Cytotoxicity

**Figure 10:**
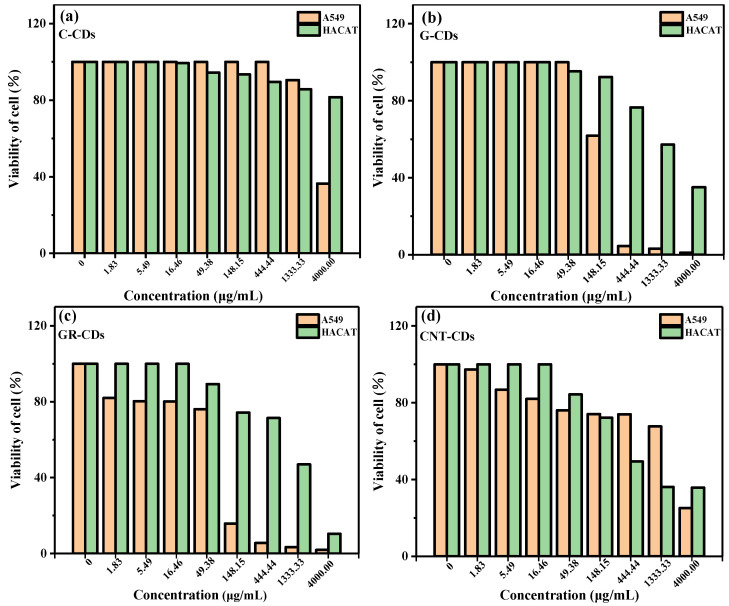
Cell cytotoxicity test of C-CDs (**a**), G-CDs (**b**), GR-CDs (**c**), and CNT-CDs (**d**) of different concentrations on A549 and HACAT cells.

Spurred by the high photothermal conversion efficiency of the CDs, we further tested their cytotoxicity with MTT assay. In this study, A549 and HACAT cells were treated with varied concentrations of CDs, and the results are presented in Figure 10. From the results, we could see that none of the CDs had cytotoxicity towards either the A549 or HACAT cells after 48 h of cultivation at low concentrations. However, after the concentrations of the CDs reached a certain threshold, the samples began to demonstrate a certain degree of cytotoxicity and the viabilities of the A549 and HACAT cells decreased in varying degrees. For instance, the viability of the A549 cells was not significantly affected even with 1333.33 μg/mL of C-CDs (Figure 10a), demonstrating the high biocompatibility of the C-CDs. On the other hand, the viabilities of the A549 cells could be maintained at a normal value with G-CDs concentrations up to 49.38 μg/mL (Figure 10b). Unfortunately, both the GR-CDs and CNT-CDs demonstrated some degree of cytotoxicity towards A549 cells at high concentrations (Figure 10c,d), which might be attributed to the exposure of large areas of graphene-like structures to A549 cells, since graphene is known to have a high cytotoxicity [40].

The trends of cytotoxicity of the CDs towards the HACAT cells were quite similar to those demonstrated for theA549 cells, but with a much higher threshold of concentrations. Overall, none of the CDs demonstrated an obvious cytotoxicity towards the HACAT cells until their concentrations were over 49.38 μg/mL (Figure 10, green columns). Specifically, the C-CDs demonstrated an extremely low cytotoxicity towards the HACAT cells; cell viability was still over 80% even when the concentration of C-CDs reached 4000 μg/mL (Figure 10a). As in the case for the A549 cells, the G-CDs had a better biocompatibility towards the HACAT cells (Figure 10b) than the GR-CDs and CNT-CDs (Figure 10c,d). In general, the cytotoxicity of the four samples was ranked as follows: C-CDs < CNT-CDs < G-CDs < GR-CDs; this may be due to the size differences between CDs. According to previous literature reports, smaller carbon materials showed higher cytotoxicity. On the other hand, the functional groups on the surface of carbon materials may also be the influencing factors of cytotoxicity [41]. In summary, we have successfully synthesized CDs with a low toxicity which are comparable to those reported in the literature [42,43,44]. Collectively considering their high photothermal conversion efficiencies and low cell cytotoxicity, the four CDs prepared in this study demonstrated high potential as photothermal therapy agents.

## 3. Materials and Methods

### 3.1. Reagents and Materials

Carbon powder (99%) less than 100 nm in size and methyl thiazolyl tetrazolium (MTT) were purchased from Sigma-Aldrich (Shanghai, China) Trading Co., Ltd. Graphite (99.95%) approximately 1300 nm in size was purchased from Shanghai Macklin Biochemical Co., Ltd. (Shanghai, China). Multilayer graphene (95%, about 6–10 layers) with a thickness of 3.4–8 nm was purchased from Suzhou Tanfeng Graphene Technology Co., Ltd. (Suzhou, China). Multi-walled carbon nanotubes (98%) were purchased from Zhongke Times Nano Energy Tech Co., Ltd. (Chengdu, China), with a radius of 10–20 nm and a length of 20–100 nm. Sulfuric acid (H_2_SO_4_, 95–98%) and chloroform (99%) were purchased from Chengdu Cologne Chemicals Co., Ltd.; nitric acid (HNO_3_, 65–68%) was purchased from Shantou DAHAO Fine Chemicals Co., Ltd. (Shantou, China). Sodium hydroxide (NaOH, 96%) was purchased from Tianjin Fengchuan chemical reagent Technology Co., Ltd. (Tianjin China). Phenolphthalein was provided by Zhongda Teaching Instrument (Suzhou, China). Fetal bovine serum (FBS) was acquired from Capricorn Scientific (Guangzhou Tianyuan Biotechnology Co., Ltd., GuangZhou, China), while RPMI-1640, MEM culture medium, and trypsin/EDTA were purchased from Giboc (Shanghai Yuchun Biotechnology Co., Ltd., Shanghai, China). Dialysis bags were purchased from Shanghai Zhenjian Biotechnology Co., Ltd. (Shanghai, China). All the reagents were used as received without further purification, unless otherwise noted. The deionized (DI) water used in all experiments was made from a Master Touch-S laboratory ultrapure water machine (Master Touch, Shanghai, China).

### 3.2. Synthesis of CDs

CDs were prepared according to a modified version of our previously reported method [45]. Briefly speaking, 250 mg of carbon precursor (carbon power, graphite, graphene, and carbon nanotubes) was transferred to a 50 mL round bottom flask with a stir bar, and then sulfuric acid (9 mL) and nitric acid (3 mL) were added. The resulting mixture was heated in an oil bath at 110 °C under violent stirring with a condenser. After 15 h, the reaction was stopped and allowed to cool down naturally. The whole reaction mixture was then further cooled down to near 0 °C with ice water, and saturated NaOH solution was added to the reaction slowly to neutralize the unreacted acids. Following that, the reaction mixture was filtered to remove unreacted carbon precursor, and the filtration was cooled down below 0 °C to have the salts (NaNO_3_, Na_2_SO_4_) precipitated and filtered. The processes of precipitation and filtering were repeated twice until no visible salt precipitation was observed in the solution. The resulting solution was concentrated to roughly 25 mL with rotovap evaporation, and then chloroform was added to perform extractions to remove the small amount of organic impurities formed during the oxidative reactions. The resulting aqueous solution was then centrifuged at 3000 rpm for half an hour and the precipitates were removed. Then, the clear top solution was dialyzed (MWCO = 3500) against DI water for 48 h, and the solution in the dialysis bag was evaporated at 75 °C to result in the final product (black powder).

### 3.3. Characterizations

The morphology of the CDs was characterized by transmission electron microscopy (TEM) with a transmission electron microscope (JEM-2100, Tokyo, Japan) with an accelerating voltage of 200 kV and a magnification of 800K times. The atomic force microscopy (AFM) was taken by a Park NX10 (Korea). The UV–Vis absorption spectrum and fluorescence emission spectrum of the CD solutions were measured by a UV-2600 (Shimadzu, Japan) and fluorescence spectrometer F97 Pro (Shanghai Prism Technology Co., Ltd., Shanghai, China) in a 1 × 1 × 3 cm quartz cuvette. The frequency-domain lifetime was measured by a FLS10000 fluorescence lifetime spectrometer (Edinburgh, UK). Fourier transform infrared (FT-IR, Middle Age Walker, Thermo, Waltham, KS, USA) spectra were recorded by a Thermo Fisher Scientific Nicolet iS50. X-ray photoelectron spectroscopy (XPS, Thermo Fisher Scientific, Waltham, KS, USA) was characterized using a K-Alpha+ with a Mono Al Kα X-ray source, with a hν = 1486.6 eV photon energy operated at 6 mA × 12 kV. X-ray powder diffraction (Rigku XRD, Japan) was conducted using a Rigaku TTRIII polycrystal X-ray diffraction equipped at 18 KW with Cu Kα radiation (λ = 1.5406 nm). Raman spectra were recorded by inVia (Renishawin, UK) equipment and zeta potential measurements were determined by Zetasizer Nano Series (Malvern, UK) equipment. The thermogravimetric analysis (TGA) data were obtained from a DSC-1600 LF synchronous thermal analyzer (Mettler, New York, NY, USA).

### 3.4. Fluorescence Stability of CDs

To study the fluorescence stability of the CDs, each solution (0.5 mg/mL) of the four CDs was placed in a lab under ambient conditions (i.e., room temperature, ambient light). The fluorescence spectra of these solutions under 460 nm excitation were then recorded and monitored for evaluation. Spectra were taken every week in the first month, and every other week in the following month.

### 3.5. Fluorescence Lifetime of CDs

The fluorescence lifetimes of the CDs were measured in aqueous solution at room temperature with an excitation wavelength set at 475 nm using a fluorescence lifetime spectrometer and all curves were best fitted to the following double exponential function:

(1) $$I=\exp\left(\frac{-t}{\tau_{1}}\right)+a_{2}\exp\left(\frac{-t}{\tau_{2}}\right)$$

where *I* is the intensity of the sum of single exponential decay, both *a*_1_ and *a*_2_ are exponential factors, and *τ*_1_ and *τ*_2_ are the decay times of the PL lifetime. Fitted results are presented in Figure S1: four samples demonstrated similar decay profiles. Then, the average fluorescence lifetimes of the four samples were calculated according to the following formula:

(2) $$\tau_{ave}=\frac{a_{1}\tau_{1}^{2}+a_{2}\tau_{2}^{2}}{a_{1}\tau_{1}+a_{2}\tau_{2}}$$

### 3.6. Determination of Carboxyl Contents of CDs

To estimate the carboxyl contents on the surfaces of the four CDs, a series of titration experiments were carried out [46]. Briefly, 2.5 mg of CDs was transferred to a conical flask, and then 25 mL of DI water was added. The mixture was ultrasonicated to result in a clear CD solution with a concentration of 0.1 mg/mL. The CD solution was then titrated by NaOH solution (0.01 M) with two drops of phenolphthalein solution as the indicator. During the titration, the conical flask was continuously shaken, and the volume of NaOH solution (*V*_NaOH_) consumed was recorded when the CD solution turned red and did not fade for half a minute. The carboxyl contents (in mmol/g) of the four CDs were then calculated following the equation below:

(3) $$C_{\mathrm{COOH}}=\frac{0.01V_{\mathrm{NaOH}}}{2.5}$$

where *C*_COOH_ is the amount of carboxyl groups in mmol found in each gram of CDs (mmol/g); the specific data for the calculations are presented in Table S2.

### 3.7. Photothermal Conversion Efficiency of CDs

To evaluate the photothermal capabilities of the CDs, a series of aqueous dispersions of the four samples with different concentrations (0.2, 0.4, 0.6, 0.8, 1.0 mg/mL) were prepared. Each of the dispersions was then irradiated continuously by an 808 nm NIR laser (2.5 W/cm^−2^) for 10 min, after which the laser was turned off and the solutions were allowed to cool naturally to room temperature. In these tests, DI water was applied as a negative control. During the whole process, the temperature was monitored and recorded by an intelligent digital photothermal camera (FLIR E4, Fengcheng Technology Co., Ltd., Shenzhen, China) every 30 s. The photothermal conversion efficiency (η) of the four samples was then calculated according to the following equation [47]:

(4) $$\eta =\frac{\mathrm{hS}\left(T_{max}-T_{surr}\right)-Q_{\mathrm{Dis}}}{I\left(1-10^{-A_{\lambda }}\right)}$$

where h is the heat transfer coefficient, S is the surface area of the quartz cuvette in cm^−2^, *T_max_* is the maximum temperature of the solution in °C, *T_surr_* is the ambient temperature in °C, Q_Dis_ is the heat absorbed by the solvent in J, I is the laser power in W cm^−2^, and A_λ_ is the absorbance of the CDs at 808 nm [48,49,50]. The A_λ_ of the C-CDs, G-CDs, GR-CDs, and CNT-CDs at 808 nm was 0.053, 0.035, 0.099, and 0.165, respectively. In the above equation, *hS* is unknown and could not be directly measured. In order to obtain its value, the following equations were derived:

(5) $$\tau_{s}=\frac{m_{D}C_{D}}{hS}$$

(6) $$t=-\tau \ln\left(\theta \right)$$

(7) $$\theta =\frac{T-T_{surr}}{T_{max}-T_{surr}}$$

where *m_D_* and *C_D_* represent the solutions mass (1 g) and heating capacity (4.2 J g^−1^) of water, respectively, *T* represents the solution temperature (°C), and *τ_s_* is derived from the linear time data and −ln(*θ*), as shown in Figures S5–S8.

### 3.8. Cytotoxicity Tests

#### 3.8.1. Cell Resuscitation

The cells were quickly removed from the liquid nitrogen tank and cultured in a water bath at a constant temperature (37 °C). Cryopreservation solution was transferred to a 15 mL centrifuge tube, and then complete culture medium was added at a ratio of 1:3 and centrifuged at 800 rpm for 5 min. Following that, the supernatant was discarded, 5 mL of complete medium was added to re-suspend the cells, and the solution was transferred to the medium. The cells were cultured in a 25 cm^2^ cell culture flask and placed in the cell incubator (saturated humidity, 37 °C and 5% CO_2_).

#### 3.8.2. Cell Passage

When the cell density reached 75–85%, the cells were sub-cultured. The original culture medium in the culture bottle was separated and discarded, and the cells were then washed with sterile PBS, which was also discarded. After that, 0.5 mL of 0.25% trypsin digested cell sand was placed in the cell incubator to digest for about 2 min (HACAT cells digest for 10 min); the digestion of the cells was monitored under a microscope. When the digestion of the cells was finished, complete medium was added to terminate the digestion, following which the cells were gently blown to form a single cell suspension. An appropriate amount of cell suspension was then taken for continued culture in a new culture bottle for standby.

#### 3.8.3. Cell Count

The cell counting plate and cover slide were washed with distilled water and dried in the air. The cover slide was then placed on the counting hole of the counting board. The cells were mixed with trypan blue in a volume ratio of 1:1. Then, 10 μL of the mixed suspension was dropped on both sides of the junction between the cell counting plate and the cover glass slide. The cells were counted with 10× observation under an objective microscope.

#### 3.8.4. MTT Assay

MTT assay was applied to detect the toxicity or inhibition of the CDs on cell proliferation. Briefly, cells in the logarithmic growth phase were digested and the number of cells was adjusted to 2500~3000 cells/well. The cells were then evenly inoculated into a 96-well cell culture plate (100 μL/well) and cultured overnight in an incubator (37 °C, 5% CO_2_) until they adhered to the wall. Twenty-four hours later, each group of the cells was added to CD solutions of different concentrations (1.83, 5.49, 16.46, 49.38, 148.15, 444.44, 1333.33, and 4000.00 ug/mL) according to the gradient concentration set in the experiment, with three pores in each group. After continuous treatment for 72 h, MTT solutions (10 mg/μL) were added into each well and continued to incubate for 4 h, after which the reaction was terminated, the culture medium was discarded, and 100 μL of DMSO was added into each well. The wells were then oscillated at medium and low speed for 15 min for dissolution and crystallization, following which the OD values of each well were measured (wavelength: 570 nm).

## 4. Conclusions

In summary, four different CDs were successfully prepared via the acid oxidation method using CNPs, graphite, graphene, and CNTs as carbon precursors, and systematic characterizations were carried out to study and compare the morphological, optical, chemical, and structural properties of CDs. Optical properties such as UV–Vis absorptions and fluorescence emissions and the types of surface functionalities of the four samples were quite similar, presumably due to their being synthesized using the same method and originating from similar precursors. However, their sizes, morphologies, and chemical compositions (i.e., carbon oxygen ratio, contents of functionalities) varied and were believed to be influenced by the subtle differences of their carbon precursors. Furthermore, the core structures of the CDs heavily resembled the intrinsic structural features of the precursors. The study shows that in the top-down approach, it might be feasible to obtain CDs with desired properties and structures by the selective adoption of carbon precursors and fine-tuning of the synthesis methods. We believe this study can provide some insights into the synthesis and understanding of top-down CDs derived from similar precursors. Furthermore, these delicately controlled differences of CDs should have an impact on their applications. For example, CNT-CDs have a large number of carboxyl groups on the surface, which makes them more suitable for drug carriers than the other three. All four of the studied CDs demonstrated very high biocompatibility and excellent photothermal conversion efficiencies, which should find potential applications for photothermal therapy. Meanwhile, due to their ideal photothermal conversion efficiency, our CDs might also find some potential applications in energy- and environment-related fields such as sunlight-assisted seawater desalination.

## Figures and Tables

**Table 1:** Optimization of reaction parameters for CDs preparation.

	CDs ^a^	Mass (mg)	H_2_SO_4_ (mL)	HNO_3_ (mL)	Temperature (°C)	Time (h)	Yield ^b^ (%)
1		250	6	6	110	15	41
2		250	8	4	110	15	45
3		250	9	3	110	15	43
4		250	3	9	110	15	40
5	C-CDs	250	12	0	110	15	0
6		250	9	3	140	15	43
7		250	9	3	110	7.5	34
8		250	9	3	110	20	47
9		500	18	6	110	15	15
10	G-CDs	250	9	3	110	15	15
11	GR-CDs	250	9	3	110	15	21
12	CNT-CDs	250	9	3	110	15	32

^a^ C-CDs, G-CDs, GR-CDs, and CNT-CDs are CDs derived from CNPs, graphite, graphene. and CNTs, respectively. ^b^ The CDs yields were calculated according to the following equation: Yield = Mass_(CDs)_/Mass_(Precursor)_ × 100%.

**Table 2:** Comparison of C/O contents, surface carboxyl groups, and zeta potential of the CDs.

		C-CDs	G-CDs	GR-CDs	CNT-CDs
1 ^a^	C (%)	66.5	61.6	54.2	56.6
2 ^a^	O (%)	33.5	38.4	45.8	43.4
3	-COOH (mmol/g)	6.5	4.3	4.4	8.5
4	Zeta potential (eV)	−5.18	−11.78	−5.93	−36.50

^a^ Relative contents after subtracting other trace impurities.

## Data Availability

The data presented in this study are available within the article and supplementary material.

## Supplementary Materials

The following supporting information can be downloaded at: https://www.mdpi.com/article/10.3390/ijms23031456/s1.
