# Supplementary material for: Synthesis Mechanisms, Structural Models, and Photothermal Therapy Applications of Top-Down Carbon Dots from Carbon Powder, Graphite, Graphene, and Carbon Nanotubes
Source: Int J Mol Sci. 2022 Jan 27;23(3):1456. doi: 10.3390/ijms23031456 (PMC8835929; doi:10.3390/ijms23031456)
Supplement: Supplementary file 1 [file ijms-23-01456-s001.zip › ijms-1552974-supplementary.pdf]

Supplementary Materials

Synthesis Mechanisms, Structural Models and Photothermal Therapy Applications of Top-Down Carbon Dots from Carbon Powder, Graphite, Graphene and Carbon Nanotubes

Wenquan Shi,[a,b,+] Qiurui Han,[a,+] Jiajia Wu,[a] Chunyu Ji,[a] Yiqun Zhou,[c] Shanghao Li,[d] Lipeng Gao,[a] Roger M. Leblanc[c] and Zhili Peng,[*a,b]

[a]School of Materials and Energy, Yunnan University, Kunming 650091, People's Republic of China.

[b]Advanced Computing Center, Materials Genome Institute, Yunnan University, Kunming 650091, P. R. China

[c]Department of Chemistry, University of Miami, 1301 Memorial Drive, Coral Gables, Florida 33146, United States.

[d]MP Biomedicals, 9 Goddard, Irvine, CA 92618, USA.

***Corresponding authors:**

(Z. P.) Tel.: +86–871–65037399; E–mail: zhilip@ynu.edu.cn.

[+]These authors contributed equally to this work.

Keywords:

Carbon dots; carbon nano powders; graphite; graphene; carbon nano tubes; photothermal therapy

Table S1. Effects of reaction temperature, time and scale on the yields of GR-CDs

	Mass (mg)	H_2SO_4 (mL)	HNO_3 (mL)	Temperature (°C)	Time (h)	Yield (%)
1	250	9	3	110	7.5	10
2	250	9	3	75	15	3
3	500	18	6	110	15	11

Table S2. Volumes of NaOH solution consumed for titrations of CDs and the corresponding carboxyl contents determined for each CDs

Sample	V_{NaOH} consumed (mL)	-COOH (mmoL/g)
C-CDs	1.7	6.8
	1.7	6.8
	1.5	6.0
G-CDs	1.1	4.4
	1.0	4.0
	1.1	4.4
GR-CDs	1.0	4.0
	1.2	4.8
	1.1	4.4
CNT-CDs	2.2	8.8
	2.1	8.4
	2.1	8.4

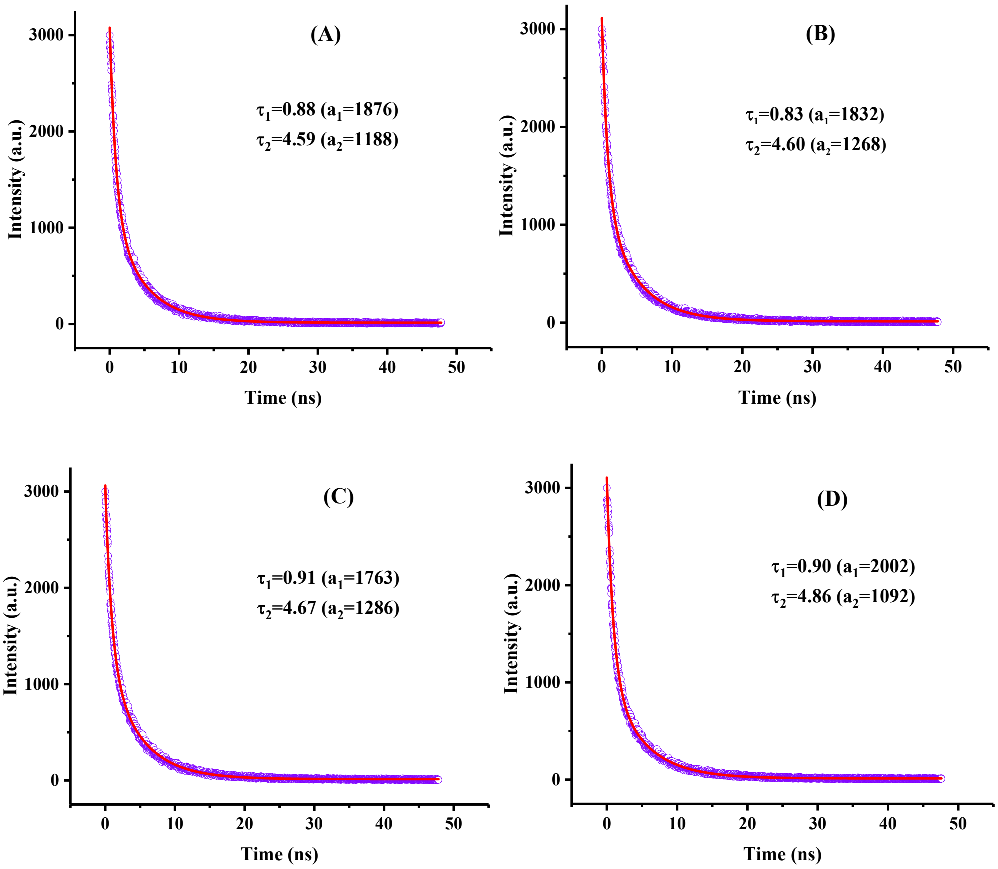

Figure S1. The photoluminescence decay curves of C-CDs (**A**), G-CDs (**B**), GR-CDs (**C**) and CNT-CDs (**D**) in 0.5 mg/mL aqueous solution, excited at 475 nm.

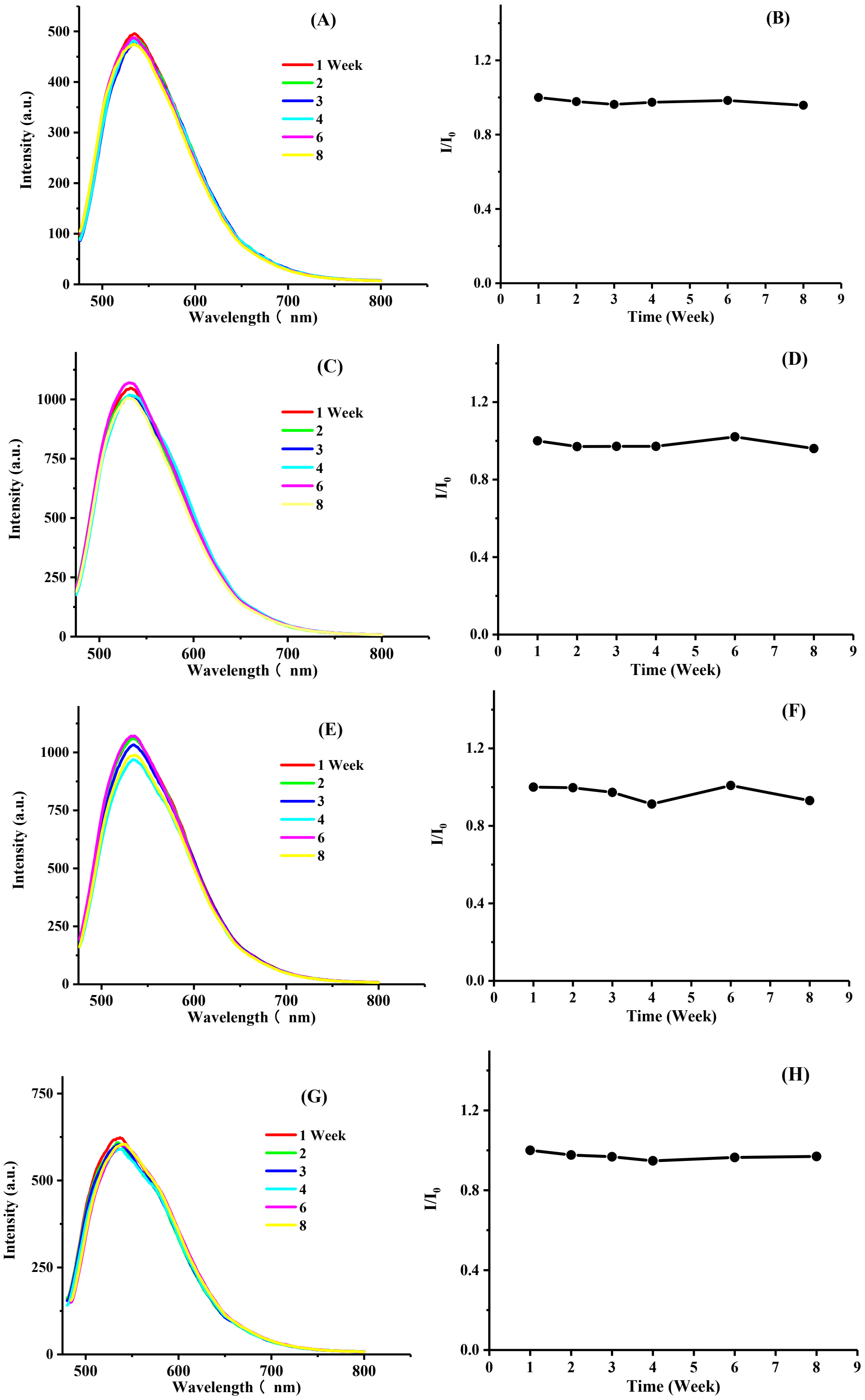

Figure S2. Photoluminescence stability tests of 0.5 mg/mL of C-CDs (**A, B**), G-CDs (**C, D**), GR-CDs (**E, F**) and CNT-CDs (**G, H**): solutions of CDs were placed in the ambient environment and their fluorescence emissions (excited at 460 nm) were monitored for two months.

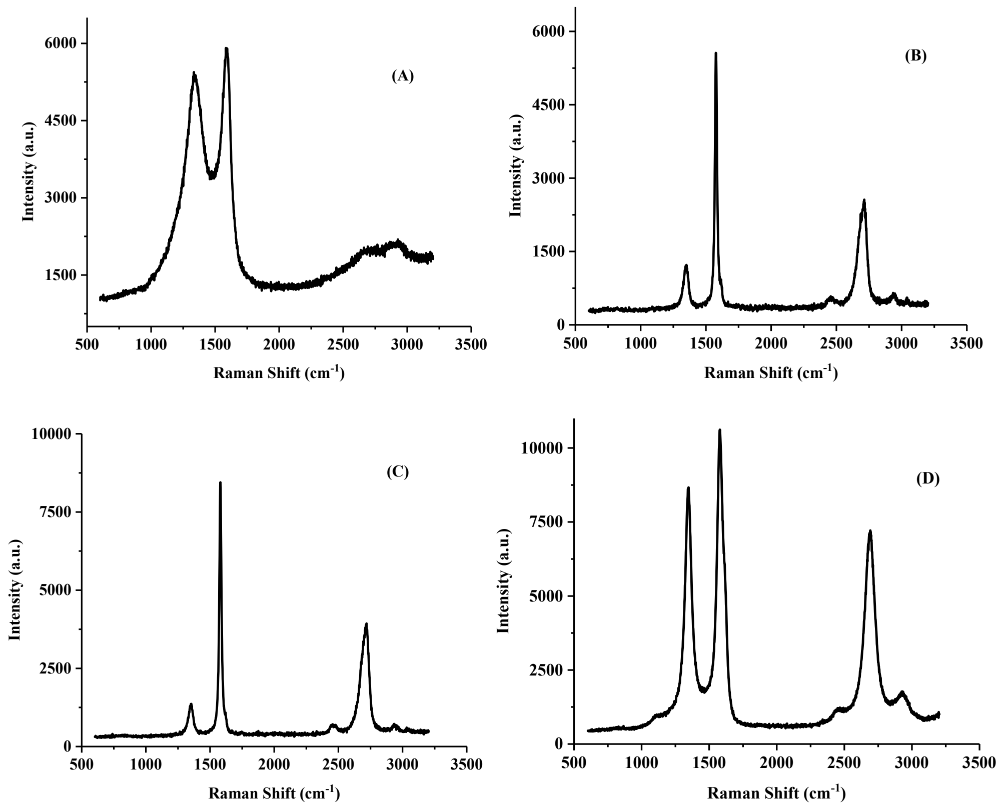

Figure S3. Raman spectra of carbon powders (**A**), graphite (**B**), graphene (**C**) and carbon nanotubes (**D**) used to synthesize CDs in this study.

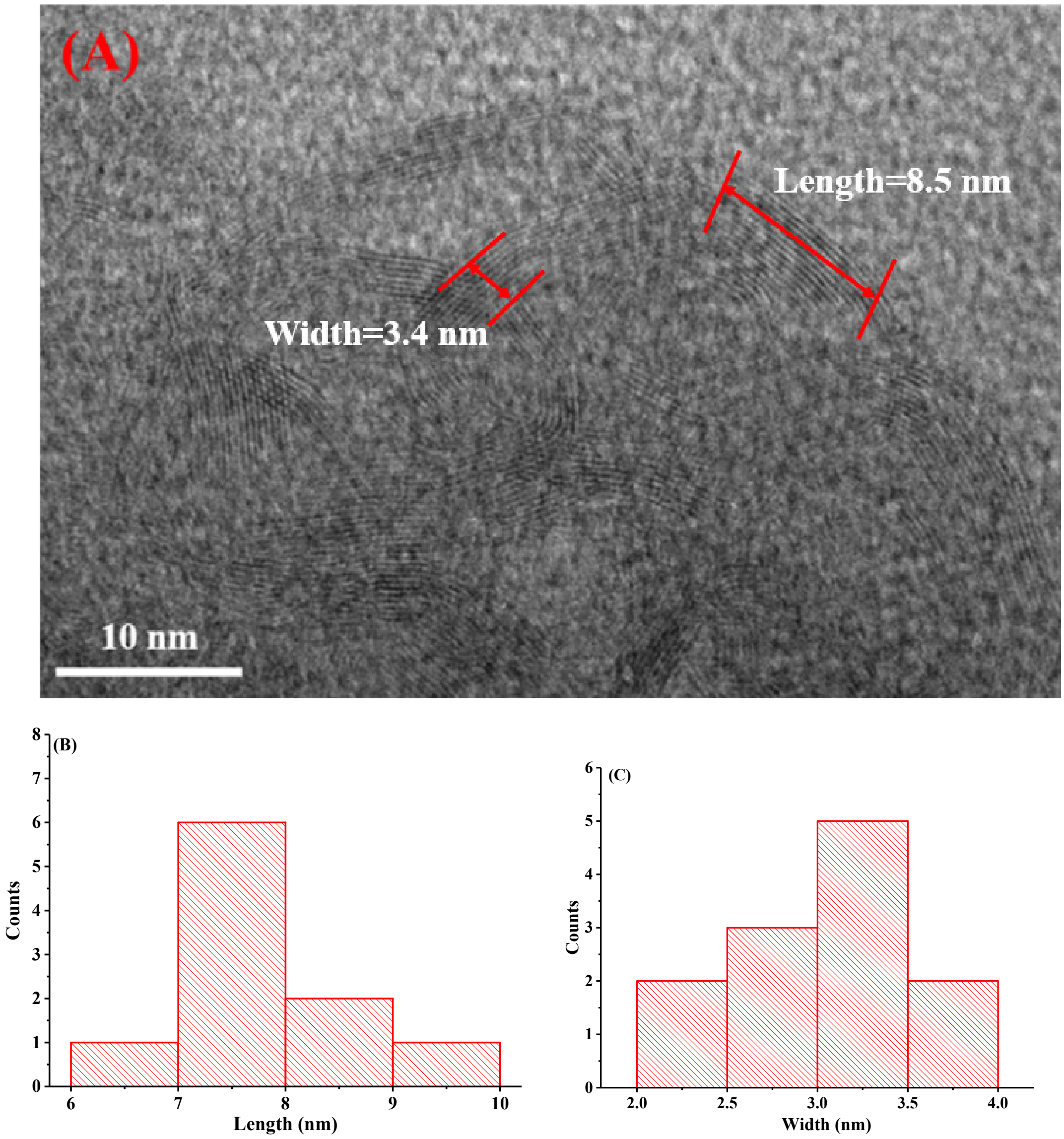

Figure S4. TEM image of broken carbon nanotubes (**A**), and the histograms showing the distributions of length (**B**) and width (**C**) of these pieces

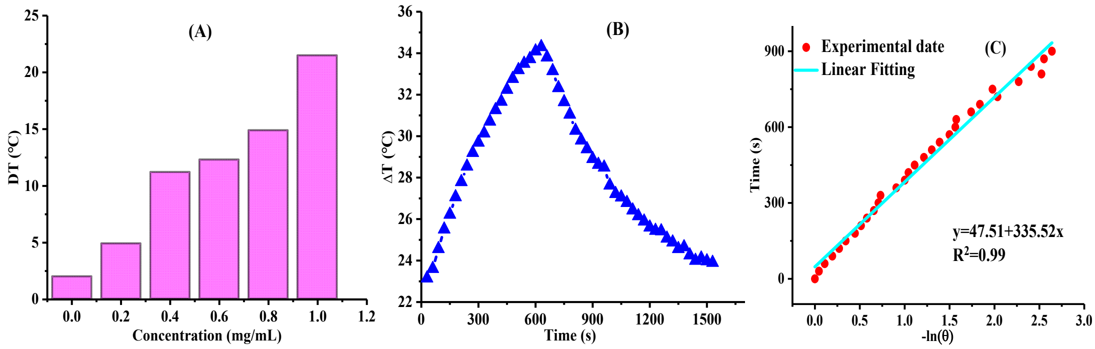

Figure S5. Photothermal properties of C-CDs: (**A**) Temperature change (ΔT) diagram of water and C-CDs solutions with concentrations range from 0.2 to 1.0 mg/mL after laser irradiation for 10 minutes; (**B**) Photothermal effect of 1 mL of C-CDs solution with concentration of 0.4 mg/mL after laser irradiation for 10 minutes; (**C**) Linear relationship between cooling time and -ln(θ) obtained from Figure S5B.

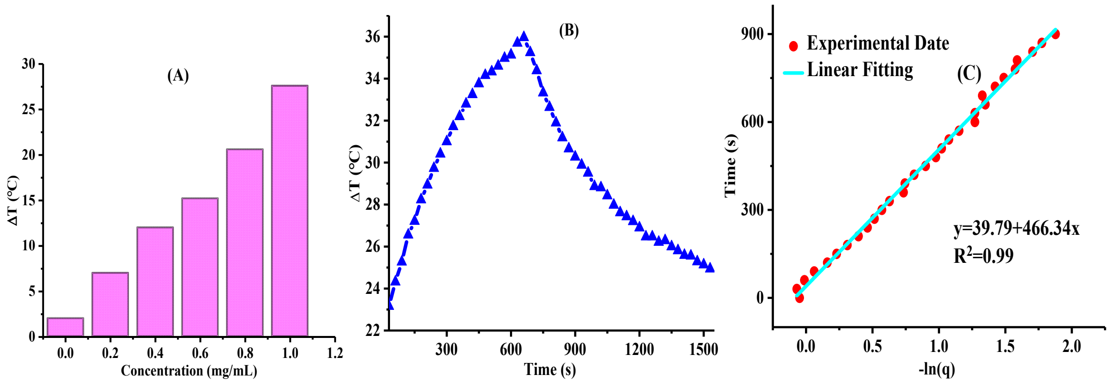

Figure S6. Photothermal properties of G-CDs: **(A)** Temperature change (ΔT) diagram of water and G-CDs solutions with concentrations range from 0.2 to 1.0 mg/mL after laser irradiation for 10 minutes; **(B)** Photothermal effect of 1 mL of G-CDs solution with concentration of 0.4 mg/mL after laser irradiation for 10 minutes; **(C)** Linear relationship between cooling time and -ln(θ) obtained from Figure S6B.

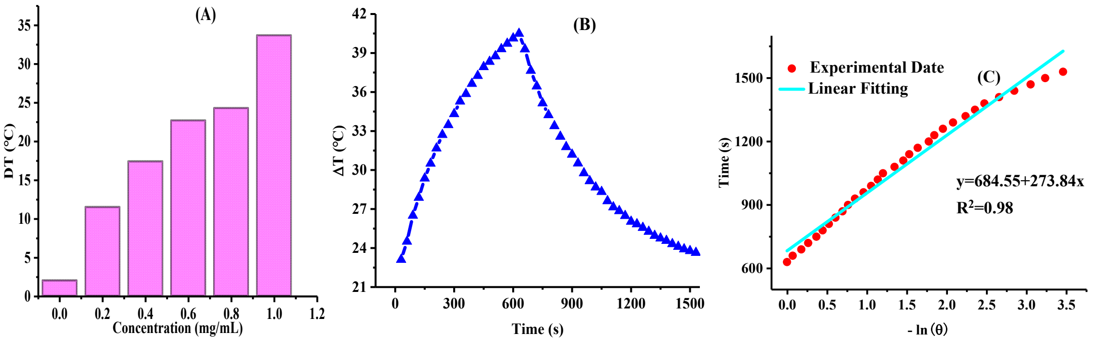

Figure S7. Photothermal properties of GR-CDs: **(A)** Temperature change (ΔT) diagram of water and GR-CDs solutions with concentrations range from 0.2 to 1.0 mg/mL after laser irradiation for 10 minutes; **(B)** Photothermal effect of 1 mL of GR-CDs solution with concentration of 0.4 mg/mL after laser irradiation for 10 minutes; **(C)** Linear relationship between cooling time and -ln(θ) obtained from Figure S7B.

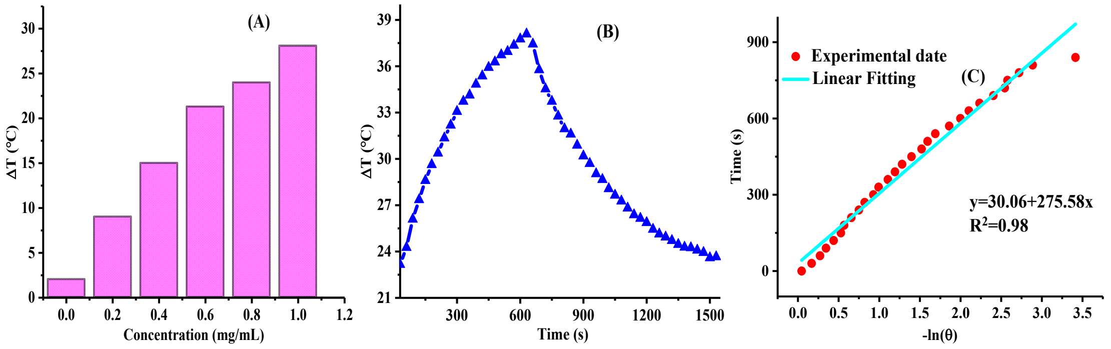

Figure S8. Photothermal properties of CNT-CDs: **(A)** Temperature change (ΔT) diagram of water and CNT-CDs solutions with concentrations range from 0.2 to 1.0 mg/mL after laser irradiation for 10 minutes; **(B)** Photothermal effect of 1 mL of CNT-CDs solution with concentration of 0.4 mg/mL after laser irradiation for 10 minutes; **(C)** Linear relationship between cooling time and -ln(θ) obtained from Figure S8B.
